# Genetic and animal model analyses reveal the pathogenic role of a novel deletion of *RELN* in schizophrenia

**DOI:** 10.1038/s41598-018-31390-w

**Published:** 2018-08-29

**Authors:** Akira Sobue, Itaru Kushima, Taku Nagai, Wei Shan, Takao Kohno, Branko Aleksic, Yuki Aoyama, Daisuke Mori, Yuko Arioka, Naoko Kawano, Maeri Yamamoto, Mitsuharu Hattori, Toshitaka Nabeshima, Kiyofumi Yamada, Norio Ozaki

**Affiliations:** 1 Department of Neuropsychopharmacology and Hospital Pharmacy, Nagoya University Graduate School of Medicine, Nagoya, Aichi Japan; 2 Department of Psychiatry, Nagoya University Graduate School of Medicine, Nagoya, Aichi Japan; 3 Institute for Advanced Research, Nagoya University, Nagoya, Aichi Japan; 4 Department of Biomedical Science, Graduate School of Pharmaceutical Sciences, Nagoya City University, Nagoya, Aichi Japan; 5 Brain and Mind Research Center, Nagoya University, Nagoya, Aichi Japan; 6 Center for Advanced Medicine and Clinical Research, Nagoya University Hospital, Nagoya, Aichi Japan; 7 Institutes of Innovation for Future Society, Nagoya University, Nagoya, Aichi Japan; 8 Advanced Diagnostic System Research Laboratory Fujita Health University, Graduate School of Health Sciences, Toyoake, Aichi Japan; 9 Aino University, Ibaraki, Osaka Japan

## Abstract

Reelin protein (RELN), an extracellular matrix protein, plays multiple roles that range from embryonic neuronal migration to spine formation in the adult brain. Results from genetic studies have suggested that *RELN* is associated with the risk of psychiatric disorders, including schizophrenia (SCZ). We previously identified a novel exonic deletion of *RELN* in a patient with SCZ. High-resolution copy number variation analysis revealed that this deletion included exons 52 to 58, which truncated the RELN in a similar manner to the *Reln* Orleans mutation (*Reln*^*rl-Orl*^). We examined the clinical features of this patient and confirmed a decreased serum level of RELN. To elucidate the pathophysiological role of the exonic deletion of *RELN* in SCZ, we conducted behavioral and neurochemical analyses using heterozygous *Reln*^*rl-Orl/*+^ mice. These mice exhibited abnormalities in anxiety, social behavior, and motor learning; the deficits in motor learning were ameliorated by antipsychotics. Methamphetamine-induced hyperactivity and dopamine release were significantly reduced in the *Reln*^*rl-Orl/*+^ mice. In addition, the levels of GABAergic markers were decreased in the brain of these mice. Taken together, our results suggest that the exonic deletion of *RELN* plays a pathological role, implicating functional changes in the dopaminergic and GABAergic systems, in the pathophysiology of SCZ.

## Introduction

Reelin protein (RELN), an extracellular matrix protein, is mainly secreted by Cajal-Retzius cells, and it plays an important role in embryonic neuronal migration and the development of the laminar structure of the cerebral cortex^1–3^. In the adult brain, RELN is produced by γ-aminobutyric acid (GABA)-ergic interneurons, and it plays a role in synaptic plasticity, dendritic morphology, and cognitive function^4–6^. The Reelin signaling pathway in the brain involves the binding of secreted RELN to its receptors, very-low-density-lipoprotein receptor (VLDLR) and apolipoprotein E receptor 2 (APOER2), inducing the phosphorylation of intracellular adaptor protein Disabled-1 (DAB1)^7,8^. Results of genetic studies have suggested that the Reelin gene (*RELN*) is associated with psychiatric disorders, including schizophrenia (SCZ) and autism spectrum disorder^9,10^. Specifically, rare variants of *RELN* have been identified as risk factors for SCZ, including *de novo* or rare missense variants^11,12^ and an exonic deletion of *RELN*^13^. We also recently identified a novel exonic deletion of *RELN* in a Japanese patient with SCZ^14^. In addition, postmortem studies have revealed that the expression of RELN is reduced at multiple brain regions in patients with SCZ^15–17^.

The roles of RELN have been studied using several lines of *Reln* mutant mice. Jackson reeler (*Reln*^*rl*−*J*^) homozygous mice carry a 150-kb genomic deletion of *Reln*, whereas Orleans reeler (*Reln*^*rl-Orl*^) homozygous mice carry an insertion of an L1 retrotransposon in exon 61 of *Reln*, resulting in the skipping of the exon in Reelin mRNA and a frameshift^18^. Additionally, mice lacking the C-terminal region of *Reln* (ΔC-KI mice) have recently been generated as a psychiatric disorder model^19^. At the protein level, *Reln*^*rl*−*J*^ does not produce Reelin protein, while *Reln*^*rl-Orl*^ produces a truncated Reelin protein lacking a part of Reelin repeat 8 and the C-terminal region^20^. Heterozygous *Reln*^*rl*−*J/*+^ mice exhibited increased anxiety, deficits in sensorimotor gating and memory^4,21,22^ as well as alterations of spine density and synaptic plasticity^4,23^. In contrast, *Reln*^*rl-Orl*^ mice have not yet been well studied.

In the present study, we examined the boundaries of the exonic deletion of *RELN* that was previously identified in a patient with SCZ^14^. The deletion included exons 52 to 58, which truncated RELN in a similar manner to the Orleans *Reln* mutation. We performed behavioral and neurochemical analyses using heterozygous *Reln*^*rl-Orl/*+^ mice. These mice showed abnormalities in anxiety, social behavior, and motor learning; the deficits in motor learning were ameliorated by antipsychotics. We also found alterations in the dopaminergic and GABAergic neuronal systems. These results suggest that the exonic deletion of *RELN* plays a pathological role, implicating functional changes in the dopaminergic and GABAergic systems, in the pathophysiology of SCZ.

## Results

### Detailed analysis of the exonic deletion of *RELN*

**Figure 1:**
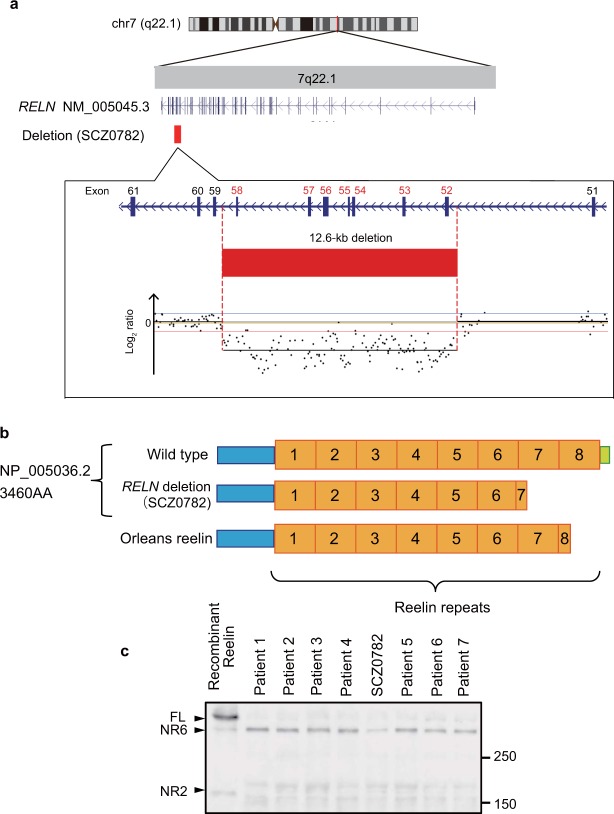
Detailed analysis of the exonic deletion in *RELN*. (**a**) The genomic coordinate of the exonic deletion is chr7:103491184–103503783. This deletion includes exons 52 to 58 of NM_005045.3. aCGH data are shown in a magnified view. (**b**) Schematic representation of the mutant RELN. The deletion corresponds to amino acid residues 2759 to 3148 (NP005036.2) within Reelin repeats 7 and 8. (**c**) Western blotting analysis of RELN in human serum. Recombinant RELN from HEK293T cells was included as a reference (lane 1). Arrowheads indicate full-length RELN (FL; 430 kDa), the C-t site cleaved form (NR6; 330 kDa), and the N-t site cleaved form (NR2; 160 kDa). In human serum, FL and NR6, but not NR2, were detected using an anti-RELN 142 antibody. In the serum of SCZ0782, the amount of NR6 was lower than that in the serum of other patients. Molecular mass markers (kDa) are indicated on the right side of the panel. This blot is cropped from the full-length blot presented in Supplementary Fig. S2.

High-resolution copy number variation (CNV) analysis revealed that the genomic coordinate of the exonic deletion of *RELN* in the SCZ patient (SCZ0782) was chr7:103491184–103503783 (12.6 kb; Fig. 1a). This deletion included exons 52 to 58 of NM_005045.3, which corresponded to amino acid residues 2759 to 3148 (NP005036.2) within Reelin repeats 7 and 8 (Fig. 1b). As this deletion was out of frame, the mutant RELN was predicted to lack amino acid residues 2759 to 3460.

### Clinical characteristics of the SCZ patient with the exonic deletion of *RELN*

The patient (SCZ0782) was a 58-year-old Japanese male with a family history of SCZ, as his sister also had SCZ. His developmental history during early childhood was unremarkable. He had a cheerful disposition and was a class representative in primary school. His academic performance was excellent. At the age of 15 years, he began to show bizarre behavior and violent outbursts against family members. He then became socially withdrawn. At the age of 24 years, he was seen by a psychiatrist for the first time, and was hospitalized for treatment. Due to poor adherence to treatment, he repeatedly relapsed with severe delusions and hallucinations, resulting in multiple hospitalizations. During hospital stays, his delusions and hallucinations remained unchanged despite adequate trials of antipsychotics, and his cognitive functioning progressively deteriorated. He complained of a feeling of electricity moving through his head, and exhibited disorganized speech and behavior. He had no insight into his illness. At the time of evaluation for this study at the age of 58 years, he still had prominent delusions and auditory hallucinations despite being treated with multiple antipsychotics. Based on his clinical course, he was considered to have treatment-resistant SCZ. He also showed negative symptoms (e.g., social withdrawal, apathy, poor self-care, and poverty of thought) and repetitive behavior (handwashing and checking). He spent most of his time alone and strongly refused to interact with others. He did not have any cerebellar symptoms, such as gait and balance coordination deficits.

The blood tests of this patient were unremarkable. T1-weighted magnetic resonance imaging showed atrophy of the left cerebral hemisphere, particularly in the frontal and parietal lobes (Supplementary Fig. S1). There were low-intensity areas in the right cerebral peduncle and left basal ganglia.

Supplementary Table S1 shows the results of the neuropsychological assessment of this patient. His premorbid intelligence quotient was estimated to be 85 using the Japanese version of the National Adult Reading Test^24^. His cognitive impairment at the time of the present study was confirmed with the Minimum Mental State Examination (22/30). His scores on neuropsychological tests were generally 1 to 2 standard deviations below the mean of normal controls, and were 1 standard deviation below the mean of SCZ patients.

### Decreased RELN level in the serum of the SCZ patient

In *Reln*^*rl-Orl*^ mice, a truncated Reelin protein that terminates within Reelin repeat 8 is synthesized, but not secreted^20^. Similarly, artificial truncated Reelin mutant proteins that terminate within any Reelin repeat are not secreted^25^. These observations raised the possibility that the truncated RELN in our SCZ patient was not being secreted into the extracellular space. Thus, to investigate the secretion of RELN in this patient, we performed western blotting analysis of RELN in SCZ patients with (n = 1; SCZ0782) or without (n = 7; patients 1 to 7) the exonic deletion of *RELN*. Full-length RELN (FL) and its C-t-cleaved form (NR6), but not the N-t-cleaved form (NR2), were detected using anti-RELN antibody. FL was weakly detected, whereas NR6 was strongly detected, indicating that FL was efficiently cleaved at the C-t site in human serum, as has been described previously^26,27^. The amount of NR6 in the serum of SCZ0782 was lower than that in the other patients (Figs 1c and S2). These results suggested that the truncated RELN of SCZ0782 was not secreted into the extracellular space.

### Behavioral abnormalities in *Reln*^*rl-Orl/*+^ mice

**Figure 2:**
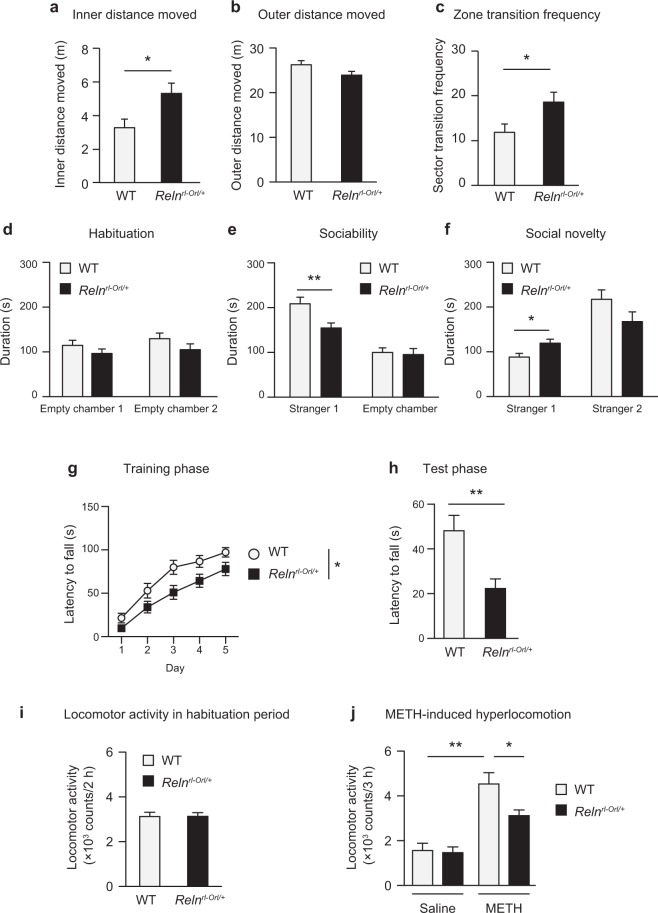
Behavioral abnormalities in *Reln*^*rl-Orl/*+^ mice. (**a**–**c**) Performance in the open field test. The distance moved in the inner (**a**) and outer (**b**) sectors, and frequency of sector transition between the inner and outer sectors (**c**). Data represent the mean ± SEM (n = 23 for WT mice; n = 28 for *Reln*^*rl-Orl/*+^ mice). *p < 0.05, significantly different from WT mice (Student’s t-test). (**d**–**f**) Performance in the social interaction test: the habituation phase (**d**) sociability (**e**) and social novelty (**f**). Data represent the mean ± SEM (n = 16 for WT mice; n = 13 for *Reln*^*rl-Orl/*+^ mice). *p < 0.05 and **p < 0.01, significantly different from WT mice (Student’s *t*-test). (**g** and **h**) Performance in the rotarod test: the training phase (**g**) and test phase (**h**). Data represent the mean ± SEM (n = 27 for WT mice; n = 28 for *Reln*^*rl-Orl/*+^ mice). *p < 0.05, significantly different from WT mice (two-way ANOVA with repeated measures). **p < 0.01, significantly different from WT mice (Student’s *t*-test). (**i** and **j**) Locomotor activity in the habituation period (**i**) and METH-induced hyperlocomotion (**j**). Data represent the mean ± SEM (n = 14 for saline-treated WT mice; n = 20 for saline-treated *Reln*^*rl-Orl/*+^ mice; n = 15 for METH-treated WT mice; n = 21 for METH-treated *Reln*^*rl-Orl/*+^ mice). *p < 0.05 and **p < 0.01, significantly different from METH-treated WT mice (Tukey’s multiple comparison test).

To examine the pathophysiological significance of the exonic deletion of *RELN*, *Reln*^*rl-Orl/*+^ mice were analyzed for the behavioral phenotype. In the open field test, the inner distance traveled by *Reln*^*rl-Orl/*+^ mice was significantly greater than that by wild-type (WT) mice (*t* (*49*) = *2*.*16*, *p* = *0*.*036*; Fig. 2a), whereas the outer distance moved was slightly less in *Reln*^*rl-Orl/*+^ mice (*t* (*49*) = *1*.*97*, *p* = *0*.*055*; Fig. 2b). *Reln*^*rl-Orl/*+^ mice crossed the border between the inner and outer sectors more frequently during the test than WT mice (*t* (*49*) = *2*.*09*, *p* = *0*.*042*; Fig. 2c), indicating a reduction in natural aversion to illuminated open areas.

Next, we evaluated sociability and social novelty preference in the three-chambered social interaction test. During the habituation phase, *Reln*^*rl-Orl/*+^ and WT mice both spent equal amounts of time exploring either of the two compartments (*genotype*: *F*_*1*,*54*_ = *3*.*14*, *p* = *0*.*082*; *chamber*: *F*_*1*,*54*_ = *0*.*93*, *p* = *0*.*338*; *interaction*: *F*_*1*,*54*_ = *0*.*07*, *p* = *0*.*793*; Fig. 2d), and there was no biased preference for the two empty chambers in either group (Fig. 2d). During the sociability phase, two-way analysis of variance (ANOVA) revealed the significant effects of genotype (*F*_*1*,*54*_ = *5*.*37*, *p* = *0*.*024*) and stranger 1 (*F*_*1*,*54*_ = *43*.*87*, *p* < *0*.*0001*) with no interaction (*F*_*1*,*54*_ = *3*.*80*, *p* = *0*.*057*; Fig. 2e). The sociability of *Reln*^*rl-Orl/*+^ mice to stranger 1 was significantly less than that of WT mice (Fig. 2e). During the social novelty preference phase, two-way ANOVA revealed the significant effects of the genotype and stranger 2 interaction (*genotype*: *F*_*1*,*54*_ = *0*.*34*, *p* = *0*.*564*; *stranger*: *F*_*1*,*54*_ = *29*.*3*, *p* < *0*.*0001*; *interaction*: *F*_*1*,*54*_ = *6*.*08*, *p* = *0*.*017*; Fig. 2f). *Reln*^*rl-Orl/*+^ mice showed a significant increase in the time spent in the chamber containing stranger 1 and less time spent in the chamber containing stranger 2 than WT mice (Fig. 2f), indicating that social approach behaviors were impaired in the *Reln*^*rl-Orl/*+^ mice.

Third, we employed the rotarod test to investigate motor coordination and motor learning. The performance of *Reln*^*rl-Orl/*+^ mice was significantly impaired over the training phase with rotation at 6 rpm (*genotype*: *F*_*1*,*53*_ = *6*.*81*, *p* = *0*.*012*; *training*: *F*_*2*,*212*_ = *77*.*48*, *p* < *0*.*0001*; *interaction*: *F*_*4*,*212*_ = *0*.*92*, *p* = *0*.*454*; Fig. 2g). In the test phase, mice were placed on a rod rotating at 12 rpm. The latency to fall from the rotarod was significantly shorter in *Reln*^*rl-Orl/*+^ mice than in WT mice (*t* (*53*) = *3*.*18*, *p* = *0*.*0024*; Fig. 2h).

There were no apparent differences in the performance of *Reln*^*rl-Orl/*+^ mice and WT mice in the elevated plus maze test, Y-maze test, novel object recognition test, and prepulse inhibition (PPI) test (Supplementary Fig. S3a–d).

Furthermore, sensitivities to methamphetamine (METH), an indirect dopaminergic agonist that stimulates dopamine release, and MK-801, a non-competitive N-methyl-D-aspartate (NMDA) receptor antagonist, were assessed in *Reln*^*rl-Orl/*+^ mice. No significant differences were observed in locomotor activity during the habituation period (*t* (*68*) = *0*.*037*, *p* = *0*.*971*; Fig. 2i). However, METH-induced hyperactivity was attenuated significantly in *Reln*^*rl-Orl/*+^ mice when compared to WT mice (*genotype*: *F*_*1*,*66*_ = *5*.*11*, *p* = *0*.*0271*; *treatment*: *F*_*1*,*66*_ = *47*.*8*, *p* < *0*.*0001*; *interaction*: *F*_*1*,*66*_ = *3*.*90*, *p* = *0*.*053*; Fig. 2j). There was no significant difference in MK801-induced hyperactivity between *Reln*^*rl-Orl/*+^ mice and WT mice (Supplementary Fig. S3e).

### Dysfunction of dopamine release in *Reln*^*rl-Orl/*+^ mice

**Figure 3:**
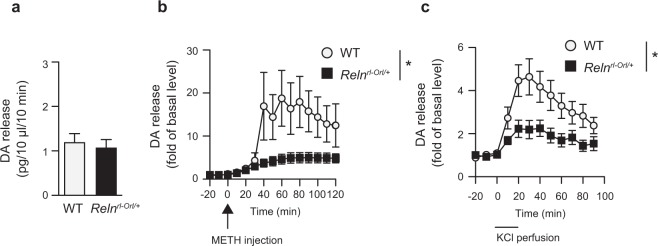
Dysfunction of dopamine release in *Reln*^*rl-Orl/*+^ mice. (**a**–**c**) Dopamine release in the NAc of *Reln*^*rl-Orl/*+^ mice measured by *in vivo* microdialysis. A dialysis probe was inserted through the guide cannula and perfused with aCSF at a flow rate of 1.0 μl/minute. Three baseline fractions were collected 3 hours after the start of the experiment. Data represent the mean ± SEM (n = 14 for WT mice; n = 14 for *Reln*^*rl-Orl/*+^ mice). (**a**) Basal release of dopamine before the stimulation. (**b**) METH-induced dopamine release in the NAc of *Reln*^*rl-Orl/*+^ mice. Following the collection of the three baseline fractions, mice were administered METH (2 mg/kg, i.p.). Data represent the mean ± SEM (n = 6 for WT mice; n = 7 for *Reln*^*rl-Orl/*+^ mice). *p < 0.05, significantly different from WT mice (two-way ANOVA with repeated measures). (**c**) Depolarization-evoked dopamine release in the NAc of *Reln*^*rl-Orl/*+^ mice. Following the collection of the three baseline fractions, high potassium-containing aCSF (60 mM KCl) was perfused for 20 minutes through the dialysis probe. Data represent the mean ± SEM (n = 8 for WT mice; n = 7 for *Reln*^*rl-Orl/*+^ mice). *p < 0.05, significantly different from WT mice (two-way ANOVA with repeated measures).

**Figure 4:**
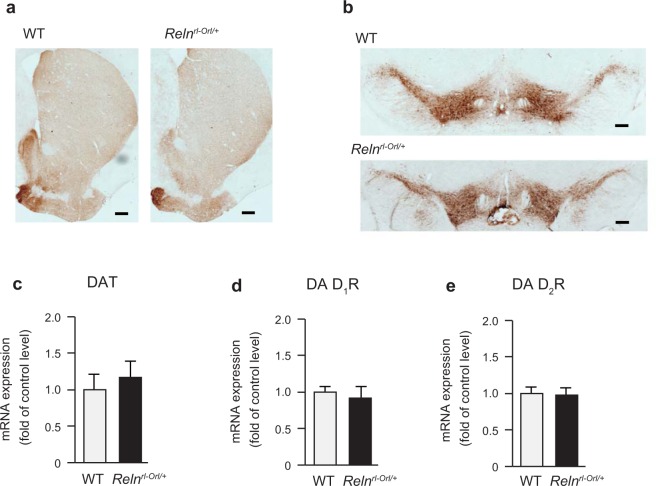
Expression analysis of dopaminergic markers in the NAc and VTA of *Reln*^*rl-Orl/*+^ mice. (**a** and **b**) TH immunostaining in the NAc (**a**) and VTA (**b**) of *Reln*^*rl-Orl/*+^ mice. (**c**–**e**) mRNA levels of *DAT* and dopamine receptors in *Reln*^*rl-Orl/*+^ mice. (**c**) *DAT* (*Slc6a3*) mRNA in the VTA. Data represent the mean ± SEM (n = 4 for WT mice; n = 4 for *Reln*^*rl-Orl/*+^ mice). (**d**) D1 receptor (*Drd1*) mRNA levels in the NAc. Data represent the mean ± SEM (n = 4 for WT mice; n = 4 for *Reln*^*rl-Orl/*+^ mice). (**e**) D2 receptor (*Drd2*) mRNA levels in the NAc. Data represent the mean ± SEM (n = 4 for WT mice; n = 4 for *Reln*^*rl-Orl/*+^ mice).

To clarify whether the reduction of METH-induced hyperactivity in *Reln*^*rl-Orl/*+^ mice was associated with alterations in dopaminergic neurotransmission in the nucleus accumbens (NAc), we examined METH-induced dopamine release by *in vivo* microdialysis. The basal release of dopamine in the NAc of *Reln*^*rl-Orl/*+^ mice did not significantly differ from that in WT mice (*t* (*26*) = *0*.*431*, *p* = *0*.*670*; Fig. 3a). The METH-induced dopamine release in the NAc was significantly lower in *Reln*^*rl-Orl/*+^ mice than in WT mice (Fig. 3b). Two-way repeated measures ANOVA revealed the significant effects of genotype (*genotype*: *F*_*1*,*11*_ = *4*.*87*, *p* = *0*.*0495*; *time after the METH injection*: *F*_*11*,*121*_ = *6*.*28*, *p* < *0*.*0001*; *time after the METH injection* × *genotype interaction*: *F*_*11*,*121*_ = *3*.*04*, *p* = *0*.*0013*; Fig. 3b). The *in vivo* microdialysis also revealed that the depolarization-evoked dopamine release in the NAc was lower in *Reln*^*rl-Orl/*+^ mice than in WT mice (*genotype*: *F*_*1*,*13*_ = *5*.*31*, *p* = *0*.*038*; *time after the KCl treatment*: *F*_*8*,*104*_ = *17*.*49*, *p* < *0*.*0001*; *time after the KCl treatment* × *genotype interaction*: *F*_*8*,*104*_ = *4*.*31*, *p* = *0*.*0002*; Fig. 3c). In contrast, no significant differences were noted between *Reln*^*rl-Orl/*+^ and WT mice in tyrosine hydroxylase (TH) immunoreactivity in the NAc and ventral tegmental area (VTA; Fig. 4a,b) nor in dopamine transporter (*DAT*; *Slc6a3*) mRNA expression levels in the VTA (Fig. 4c). The expression levels of dopamine D_1_ and D_2_ receptor mRNA were not significantly different in the NAc of *Reln*^*rl-Orl/*+^ mice and WT mice (Fig. 4d,e).

### The expression of genes encoding GABA_A_ receptors and *G*AD*67* in the brain of *Reln*^*rl-Orl/*+^ mice

**Figure 5:**
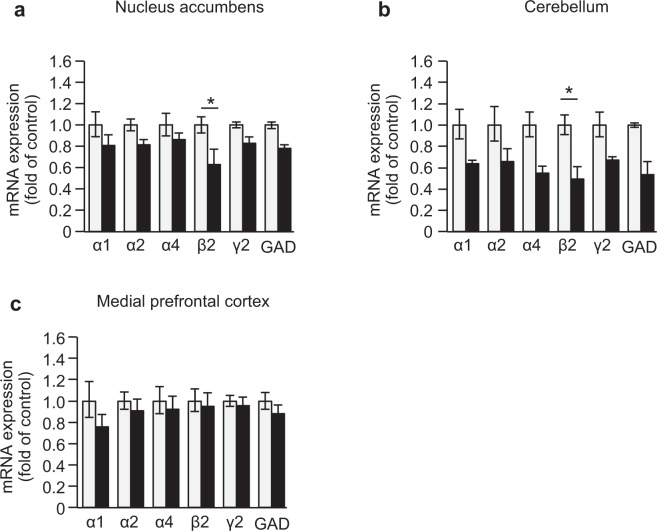
Decreases in GABA_A_ receptor subunits and *GAD* mRNA levels in the brain of *Reln*^*rl-Orl/*+^ mice. The (**a**) nucleus accumbens, (**b**) cerebellum, and (**c**) medial prefrontal cortex. The transcripts in WT and *Reln*^*rl-Orl/*+^ mice were quantified by real-time RT-PCR using the 2^−ΔΔCT^ method, and normalized to the internal reference housekeeping gene *GAPDH*. Data represent the mean ± SEM (n = 4 for WT mice; n = 3 to 4 for *Reln*^*rl-Orl/*+^ mice). *p < 0.05, significantly different from WT mice (two-way ANOVA).

A decrease in the number of GABA interneurons has been reported in SCZ patients as well as heterozygous reeler mice^28^. Accordingly, we examined the mRNA expression of genes encoding GABA_A_ receptor subunits and GABA-synthesizing enzymes (i.e., glutamic acid decarboxylase, GAD67) in the brain of *Reln*^*rl-Orl/*+^ mice. We found that the expression levels of *β2* mRNA in the NAc and cerebellum were significantly lower in *Reln*^*rl-Orl/*+^ mice than in WT mice (*NAc*: *GABAergic markers*, *F*_*5*,*36*_ = *119*.*67*, *p* < *0*.*0001*; *Genotype*, *F*_*1*,*36*_ = *20*.*6*, *p* < *0*.*0001*; *Interaction*, *F*_*5*,*36*_ = *0*.*776*, *p* = *0*.*574*; Fig. 5a. *Cerebellum*: *GABAergic markers*, *F*_*5*,*30*_ = *190*.*0*, *p* < *0*.*0001*; *Genotype*, *F*_*1*,*30*_ = *49*.*1*, *p* < *0*.*0001*; *Interaction*, *F*_*5*,*30*_ = *0*.*466*, *p* = *0*.*798*; Fig. 5b). In the medial prefrontal cortex (mPFC), no significant differences were observed in the mRNA levels of the genes encoding GABA_A_ receptor subunits and GAD67 (*GABAergic markers*: *F*_*5*,*36*_ = *25*.*5*, *p* < *0*.*0001*; *Genotype*, *F*_*1*,*36*_ = *3*.*00*, *p* = *0*.*092*; *Interaction*, *F*_*5*,*36*_ = *0*.*283*, *p* = *0*.*920*; Fig. 5c).

### Spine morphology in the brain of *Reln*^*rl-Orl/*+^ mice

**Figure 6:**
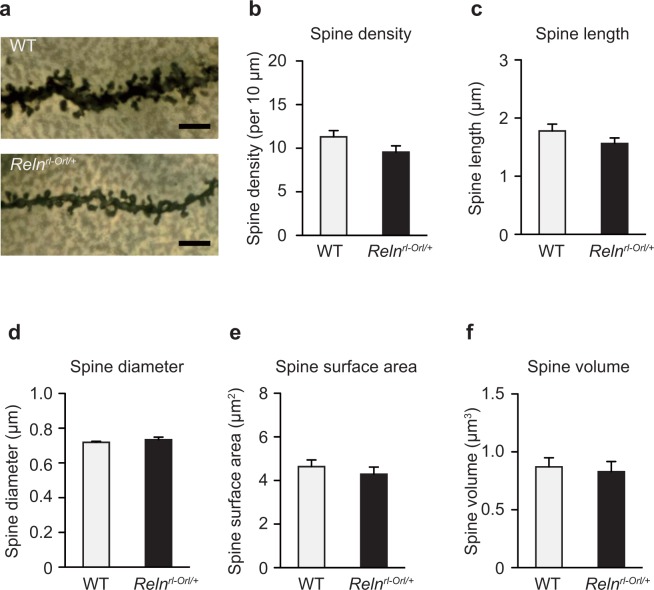
Dendritic spine analysis in *Reln*^*rl-Orl/*+^ mice. Representative images of the spines of cortical pyramidal neurons (**a**) (Golgi staining; scale bar: 5 µm). Quantitative analysis of spine density (**b**) spine length (**c**) spine diameter (**d**) spine surface area (**e**) and spine volume (**f**) of the cortical pyramidal neurons. Data represent the mean ± SEM (n = 20 neurons from four WT mice and n = 25 neurons from five *Reln*^*rl-Orl/*+^ mice in **b**–**f**).

No obvious structural abnormalities were noted in the brain of *Reln*^*rl-Orl/*+^ mice. We next analyzed the spine morphology of pyramidal neurons in layer II/III of the mPFC by Golgi staining. The dendritic spine density and length tended to be decreased in *Reln*^*rl-Orl/*+^ mice, but not to a statistically significant level (*density*: *p* = *0*.*129*, *length*: *p* = *0*.*188*; Fig. 6a–c). No significant differences were noted in the spine diameter, spine surface area, and spine volume (Fig. 6d–f).

### Effects of antipsychotics on impaired rotarod performance in *Reln*^*rl-Orl/*+^ mice

**Figure 7:**
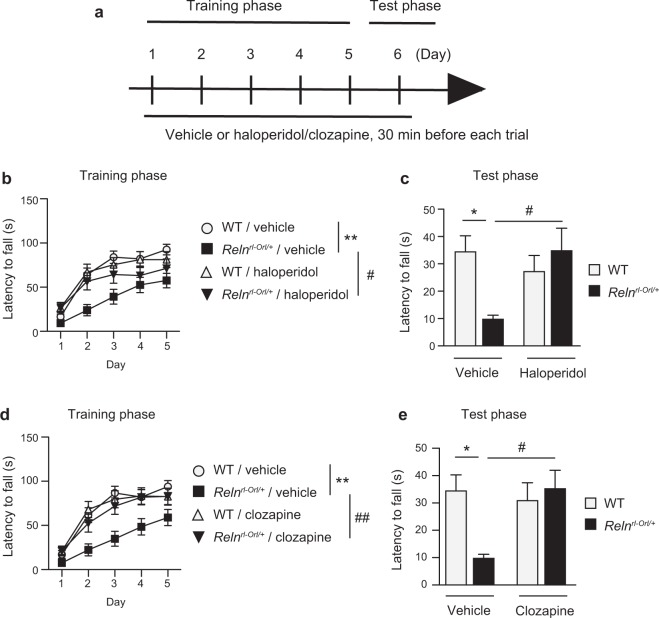
Effects of antipsychotics on impaired rotarod performance in *Reln*^*rl-Orl/*+^ mice. (**a**) Experimental protocol for the pharmacological study with haloperidol or clozapine. (**b** and **c**) Effects of haloperidol on impaired performance in the training phase (**b**) and test phase (**c**). Data represent the mean ± SEM (n = 25 for vehicle-treated WT mice; n = 24 for vehicle-treated *Reln*^*rl-Orl/*+^ mice; n = 22 for haloperidol-treated WT mice; n = 22 for haloperidol-treated *Reln*^*rl-Orl/*+^ mice). *p < 0.05 and **p < 0.01, significantly different from vehicle-treated WT mice. ^#^*p* < 0.05, significantly different from vehicle-treated *Reln*^*rl-Orl/*+^ mice. (**d** and **e**) Effects of clozapine on impaired performance in the training phase (**d**) and test phase (**e**). Data represent the mean ± SEM (n = 21 for vehicle-treated WT mice; n = 20 for vehicle-treated *Reln*^*rl-Orl/*+^ mice; n = 22 for clozapine-treated WT mice; n = 22 for clozapine-treated *Reln*^*rl-Orl/*+^ mice). *p < 0.05 and **p < 0.01, significantly different from vehicle-treated WT mice. ^#^*p* < 0.05 and ^##^*p* < 0.01, significantly different from vehicle-treated *Reln*^*rl-Orl/*+^ mice.

We examined the effects of antipsychotics on impaired rotarod performance in *Reln*^*rl-Orl/*+^ mice. The mice were administered haloperidol or clozapine 30 minutes before each training and test phase of the rotarod test (Fig. 7a). Repeated treatments with haloperidol ameliorated the impaired performance of *Reln*^*rl-Orl/*+^ mice over the training phase (*days*: *F*_*4*,*356*_ = *83*.*93*, *p* < *0*.*0001*; *genotype* × *days interaction*: *F*_*4*,*356*_ = *3*.*89*, *p* = *0*.*0042*; *haloperidol* × *days interaction*: *F*_*4*,*356*_ = *1*.*85*, *p* = *0*.*118*; *genotype* × *haloperidol* × *days interaction*: *F*_*4*,*356*_ = *1*.*42*, *p* = *0*.*226*; *genotype*: *F*_*1*,*89*_ = *9*.*87*, *p* = *0*.*0023*; *haloperidol*: *F*_*1*,*89*_ = *1*.*93*, *p* = *0*.*168*; *genotype* × *haloperidol interaction*: *F*_*1*,*89*_ = *2*.*64*, *p* = *0*.*108*; Fig. 7b). In the test phase, the latency to fall from the rotarod was significantly longer in haloperidol-treated *Reln*^*rl-Orl/*+^ mice than in vehicle-treated *Reln*^*rl-Orl/*+^ mice (*genotype*: *F*_*1*,*89*_ = *3*.*94*, *p* = *0*.*092*; *haloperidol*: *F*_*1*,*89*_ = *1*.*70*, *p* = *0*.*196*; *genotype* × *haloperidol interaction*: *F*_*1*,*89*_ = *8*.*48*, *p* = *0*.*0045*; Fig. 7c). Similar results were obtained from repeated treatments with clozapine in *Reln*^*rl-Orl/*+^ mice over the training phase (*days*: *F*_*4*,*324*_ = *97*.*23*, *p* < *0*.*0001*; *genotype* × *days interaction*: *F*_*4*,*324*_ = *3*.*70*, *p* = *0*.*0058*; *clozapine* × *days interaction*: *F*_*4*,*324*_ = *1*.*09*, *p* = *0*.*36*; *genotype* × *clozapine* × *days interaction*: *F*_*4*,*324*_ = *1*.*60*, *p* = *0*.*173*; *genotype*: *F*_*1*,*81*_ = *8*.*23*, *p* = *0*.*0053*; *clozapine*: *F*_*1*,*81*_ = *3*.*79*, *p* = *0*.*0551*; *genotype* × *clozapine interaction*: *F*_*1*,*81*_ = *4*.*63*, *p* = *0*.*0344*; Fig. 7d) and in the test phase (*genotype*: *F*_*1*,*81*_ = *2*.*77*, *p* = *0*.*0998*; *clozapine*: *F*_*1*,*81*_ = *3*.*54*, *p* = *0*.*0635*; *genotype* × *clozapine interaction*: *F*_*1*,*81*_ = *6*.*43*, *p* = *0*.*0132*; Fig. 7e). Haloperidol and clozapine had no effect on the performance of WT mice (Fig. 7).

## Discussion

Reelin is a key regulator of brain development and it acts on the first cortical structures in the central nervous system. It has also been implicated in synaptic plasticity, learning and memory^2,4,6^. Results from genetic studies have suggested that rare variants of *RELN* are associated with the risk of SCZ, including a *de novo* missense variant in a sporadic patient^9^ and a rare missense variant co-segregating with SCZ in a multiplex family^12^. We previously identified a novel exonic deletion of *RELN* in a Japanese patient with SCZ^14^. In the present study, we revealed that the deletion included exons 52 to 58, which corresponded to Reelin repeats 7 and 8. As this deletion was out-of-frame, the mutant Reelin protein was predicted to lack the region from Reelin repeat 7 to the C-terminal end. In contrast, the *Reln*^*rl-Orl*^ mutation was caused by the insertion of an L1 retrotransposon within exon 61 of *Reln* at Reelin repeat 8, leading to skipping of the exon in the Reelin mRNA and a frameshift^18^. Thus, similar to the *Reln*^*rl-Orl*^ mutation, the exonic deletion identified in the SCZ patient resulted in a truncated RELN. We observed that the RELN level in the serum of this patient was decreased. As the Reelin repeats are essential for the secretion of RELN^25^, the decreased serum RELN level may have been a result of impaired RELN secretion. Corroborating this, in *Reln*^*rl-Orl*^ mice, a truncated Reelin protein that terminates within Reelin repeat 8 was synthesized, but not secreted^20^. Interestingly, a recent study showed that *RELN* missense variants within the Reelin repeats, which were identified in patients with autism spectrum disorder, also reduced the secretion of RELN^29^.

To elucidate the pathophysiological role of the exonic deletion of *RELN* in SCZ, we performed behavioral analyses on *Reln*^*rl-Orl/*+^ mice. *Reln*^*rl-Orl/*+^ mice showed a significant increase in exploratory behavior in the inner zone of the open field and frequent zone transition between the inner and outer zones. These results suggest a reduction in natural aversion to illuminated open areas in *Reln*^*rl-Orl/*+^ mice. This reduction in anxiety-like behavior has been reported in *Reln*^*r*−*orl*^ and *Δ*C-KI mice^19,30,31^ (Table 1). In addition to these models, *Dab1* mutant mice also show reductions in anxiety-like behavior^32,33^. DAB1 receives a tyrosine phosphorylation signal from RELN^8^; as such, RELN-DAB1 signaling may be associated with anxiety. Furthermore, *Reln*^*rl-Orl/*+^ mice also exhibited some abnormalities on the social interaction test. These results indicated that *Reln*^*rl-Orl/*+^ mice have some impairments in sociability. Social dysfunction is also observed in *Δ*C-KI mice^19^ (Table 1). These results in *Reln* mutant mice are consistent with the social dysfunction observed as a symptom of SCZ^34^. Interestingly, *Reln*^*rl-Orl/*+^ mice showed some deficits in motor coordination and motor learning on the rotarod test, and the deficits were ameliorated by antipsychotics. The cerebellum is associated with motor or associative learning and the modulation of cognitive processes^35^. Additionally, patients with SCZ exhibit impaired cerebellar function^36^. Although we have examined the effects of antipsychotics on social dysfunction and cognitive impairments in *Reln*^*rl-Orl/*+^ mice, these phenotypes were difficult to evaluate in the vehicle-treated *Reln*^*rl-Orl/*+^ mice due to the influence of the stress of injection, as BALB/c mice are more sensitive to stress than other strains and show stress-induced aggressive behaviors^37^.

**Table 1:** Comparison of behavioral changes in *Reln* mutant mice.

Mutant mice strains (Background)	Sobue *et al*.^49^ (Present study)	Lalonde *et al*.^30^	Tueting *et al*.^21^	Salinger *et al*.^22^	Qiu *et al*.^4^	Sakai *et al*.^19^
	Orleans hetero (BALB/C)	Orleans homo (BALB/C)	Jackson hetero (B6C3Fe)	Jackson hetero (B6C3Fe)	Jackson hetero (B6C3Fe)	*Δ*C-KI (C57BL/6)
Age	10–15 weeks ♂♀	12 weeks ♂	9 weeks ♂	10 weeks ♂	6 weeks ♂	11 weeks ♂
Gait width	n/a	n/a	n/a	=	n/a	n/a
Nose poke	n/a	n/a	n/a	=	n/a	n/a
Stationary beams	n/a	↓	n/a	n/a	n/a	n/a
Coat-hanger test	n/a	↓	n/a	n/a	n/a	n/a
Visual cliff	n/a	n/a	n/a	=	n/a	n/a
Acoustic responsiveness	n/a	n/a	n/a	↑(Intensity response)	n/a	n/a
Olfactory guidance	n/a	n/a	n/a	=	n/a	n/a
Grip strength	n/a	n/a	n/a	n/a	n/a	=
Wire hang latency	n/a	n/a	n/a	n/a	n/a	↓
Locomotor activity	=	↑	=	n/a	n/a	n/a
Open field	↑	↑	n/a	=	=	↑
Light dark test	n/a	n/a	n/a	=	=	=
Elevated plus maze	=	↓	↓	n/a	=	↑
Tail suspension test	n/a	n/a	n/a	n/a	n/a	=
Porsolt forced swim test	n/a	n/a	n/a	n/a	n/a	=
Y-maze	=	n/a	n/a	n/a	n/a	=
Barnes maze test	n/a	n/a	n/a	n/a	n/a	=
T-maze	n/a	=	n/a	n/a	n/a	↓(Working memory)
Novel object recognition	=	n/a	n/a	↓	n/a	n/a
Social interaction test	↓	n/a	n/a	=	n/a	↓
Rotarod test	↓	↓	n/a	n/a	=	↑
Fear conditioning test	n/a	n/a	n/a	=	↓(Context)	=
Water maze test	n/a	↓	n/a	n/a	=	n/a
Hot plate test	n/a	n/a	n/a	n/a	n/a	=
Prepulse inhibition	↑(Acoustic response)	n/a	↓(80 dB 55–63 days)	=	↓(82 dB)	=
MK801-induced hyperlocomotion	=	n/a	n/a	n/a	n/a	n/a
METH-induced hyperlocomotion	↓	n/a	n/a	n/a	n/a	n/a

^↑^Higher than wild type; ^↓^lower than wild type; ^=^no difference; n/a: not applicable.

Hypersensitivity to psychostimulants has been reported in patients with SCZ^38^, and may represent a biological feature implicated in psychosis. In the present study, METH-induced hyperactivity was significantly weaker in *Reln*^*rl-Orl/*+^ mice than in WT mice. The *in vivo* microdialysis also revealed that the METH-induced and depolarization-evoked dopamine release in the NAc was lower in *Reln*^*rl-Orl/*+^ mice than in WT mice. While the sensitivity of reeler mice to psychostimulants was the opposite of that seen in patients with SCZ, this result is consistent with the phenotype of *Reln*^*rl*−*J/*+^. The *Reln*^*rl*−*J/*+^ mutation downregulates dopamine receptors and modifies their downstream molecules, i.e., intracellular proteins critical for dopaminergic neurotransmission, thereby attenuating METH-induced hyperlocomotion^39^. The *in vivo* microdialysis study suggests that presynaptic dopamine release in the NAc is impaired in *Reln*^*rl-Orl/*+^ while there was no change in TH immunoreactivity and in dopamine receptor mRNA expression in the NAc or VTA. The striatum and cortex are primary targets of the nigrostriatal and mesocortical dopamine systems, respectively^40^. Acute METH administration increases dopamine release from the VTA into the NAc and cortex^41^. METH also reverses not only monoamine transporters, but also DAT in the striatum^41,42^. Further studies are needed to confirm whether *Reln*^*rl-Orl/*+^ affects dopamine release in other brain areas, such as the striatum and cortex. In addition to functional changes in the dopaminergic system, decreases in GABAergic markers were found in the NAc and cerebellum. Previous studies have suggested that hypofunction of the GABAergic signaling system in the cerebellum contributes to the pathologies of patients with SCZ^16,43,44^.

In *Reln*^*rl*−*J/*+^ mice, the spine density and spine length were reported to be significantly decreased^23^. Similarly, our *Reln*^*rl-Orl/*+^ mice showed a non-significant decrease in spine density and spine length in the pyramidal neurons of the cortex (Fig. 6). These findings suggest that the *Reln* mutations lead to impaired synaptic transmission through a deficit in spine formation. Dendritic spines are the principal targets for synaptic transmission, and spine abnormalities have been implicated in SCZ^45,46^. Whether spine abnormalities are observed in the cerebellum should also be examined.

A detailed comparison of the phenotypes of the SCZ patient with the exonic deletion of *RELN* and *Reln*^*rl-Orl/*+^ mice revealed similarities and differences. The similarities included social dysfunction, motor learning impairments and cognitive dysfunction. The SCZ patient strongly refused to interact with others and showed severe cognitive impairments. His cognitive impairment was also confirmed by his neuropsychological assessment. The differences included the psychosis-related phenotypes: reductions in METH-induced hyperactivity due to reduced dopamine release were observed in the *Reln*^*rl-Orl/*+^ mice, whereas delusions and hallucinations, which are assumed to result from a hyper-responsive dopamine system, were observed in the patient with the exonic deletion of *RELN*. Although the precise reason for this discrepancy is unclear, it is possible that functional effects of the two mutations may not be exactly the same. For example, as Reelin repeat 7 was intact only in *Reln*^*rl-Orl/*+^ mice, this may have different functional influences on the dopamine system. Thus, *Reln*^*rl-Orl/*+^ mice may better recapitulate the social dysfunction and cognitive impairment of SCZ than the positive symptoms. Social dysfunction and cognitive impairment are also phenotypes associated with autism spectrum disorder, which has been linked to rare variants of *RELN*^47^.

In conclusion, our results suggest that the exonic deletion of *RELN* plays a pathological role, involving functional changes in the dopaminergic and GABAergic systems, in the pathogenesis of SCZ.

## Materials and Methods

### Detailed analysis of the exonic deletion of *RELN*

The exonic deletion of *RELN* was firstly identified in a patient (SCZ0782) with SCZ in our genome-wide CNV analysis^14^. In the present study, we examined the boundaries of the deletion in more detail. High-resolution array comparative genomic hybridization (aCGH) was performed using a NimbleGen custom-made fine-tiling array (90-bp probe spacing; NimbleGen, Madison, WI, USA) targeting the deletion plus the flanking regions on both sides. Using genomic DNA extracted from the blood of SCZ0782, aCGH was performed according to the manufacturer’s instructions. CNV determinations were made with Nexus Copy Number software v7.5 (BioDiscovery, El Segundo, CA, USA) using the Fast Adaptive States Segmentation Technique 2 (FASST2) algorithm, a hidden Markov model-based approach. The thresholds used to assess copy number loss were set at log2 values of −0.5. The significance threshold p-value was set at 1 × 10^−6^, and at least five contiguous probes were required for CNV calls. Genomic locations are reported in GRCh38/hg38 coordinates.

### Clinical characteristics of the SCZ patient with the exonic deletion of *RELN*

We obtained information on the clinical characteristics of the SCZ patient (SCZ0782) with the exonic deletion of *RELN* from medical records. This included the developmental history, age at onset, psychiatric symptoms, hospitalizations, doses of antipsychotics, presence of treatment resistance, and results of brain imaging and laboratory tests. This patient was administered neuropsychological tests.

This study was approved by the ethics committee of Nagoya University, and written informed consent was obtained from the subject.

### Cell culture and production of recombinant *RELN*

HEK293T cells were cultured as described previously^25^. To obtain recombinant RELN, HEK293T cells were transfected with the full-length *RELN* expression vector pCrl^48^ as described previously^25^.

### Western blotting

Western blotting was performed as described previously^25^. The protein concentration of human serum was measured using the Quick Start Bradford Protein Assay (Bio-Rad Laboratories, Hercules, CA, USA). Human serum was solubilized with sodium dodecyl sulfate sample buffer, and equal aliquots of protein (45 µg) were subjected to sodium dodecyl sulfate polyacrylamide gel electrophoresis followed by western blotting using anti-RELN antibody 142 (MAB5366, 1:1000, Millipore, Billerica, MA, USA). Images were captured using a LAS-4000 mini (Fujifilm, Tokyo, Japan).

### Animals

The *Reln* ‘Orleans’ mutation (*Reln*^*rl-Orl*^, RBRC00063) inbred strains of mice with the BALB/c background were provided by the RIKEN BioResource Center (Tsukuba, Japan). The strain was maintained in our laboratory. Heterozygous reeler (*Reln*^*rl-Orl/*+^) mice were generated by intercrossing *Reln*^*rl-Orlr-Orl/*+^ males and BALB/c females. WT BALB/c littermates were used as controls. *Reln*^*rl-Orl/*+^ and WT mice were 10 to 15 weeks old when used in the experiments. All of the animal protocols were approved by the Animal Care and Use Committee of Nagoya University Graduate School of Medicine; in addition, the Principles for the Care and Use of Laboratory Animals, which were approved by the Japanese Pharmacological Society, and the National Institutes of Health Guide for the Care and Use of Laboratory Animals were followed.

### Animals and behavioral analysis

In the open field test, mice were placed at the center of an open field (diameter, 60 cm; height, 35 cm) under a moderate light condition (60 lx) and allowed to explore for 5 minutes while their activity was recorded by using the EthoVision automated tracking program (Noldus, Wageningen, Netherlands)^49^. The open field consists of two areas, an inner area (diameter, 40 cm) and an outer area surrounding the inner area. The movement of mice was recorded via a camera mounted above the open field. Measurements of activity included distance travelled in each area.

In the social interaction test, we used the experimental paradigm described by Alkam *et al*. to measure sociability and social novelty preference behavior^50^. All sessions were conducted under a condition of illumination (15 lx). In the habituation phase, the test mouse was placed in the chamber and allowed to explore for 10 minutes. In the sociability session, an unfamiliar BALB/c mouse (stranger 1), who had no prior contact with the subject mouse, was placed in one of the side chambers. The test mouse was allowed to explore the entire social test box for a 10-minute session. In the social novelty session, a second unfamiliar mouse (stranger 2) was placed in the chamber that had been empty during the sociability test. Measurements were taken of the amount of time spent in each zone by the EthoVision automated tracking program (Noldus, Wageningen, Netherlands). A zone was defined as the area surrounding the Plexiglas cylinder (diameter of 19 cm).

In the METH-induced hyperlocomotion test, mice were injected with saline or METH (2 mg/kg intraperitoneal injection (i.p.); Sumitomo Dainippon Pharma, Osaka, Japan), and locomotor activity was measured for 180 minutes. In the measurement of locomotor activity, mice were placed individually in a transparent acrylic cage with a black frosted Plexiglas floor (25 × 25 × 20 cm), and locomotor activity was measured every 5 minutes for 60 minutes using digital counters with an infrared sensor (NS-DAS-8; Neuroscience, Tokyo, Japan). *Reln*^*rl-Orl/*+^ and WT mice were habituated to the test environment for 120 minutes before the measurement of locomotor activity (habituation period).

The rotarod test was performed according to a previous study^51^ with minor modifications. In brief, the rotarod test was performed under a moderate light condition (15 lx). Mice were trained for 5 consecutive days. During this training phase, mice were placed on a rod rotating at 6 rpm, and the time until the mouse falls from the rod was measured. If a mouse stayed on the rod until the end of the 2-minute trial, a time of 120 seconds was recorded. Subsequently, the test phase was performed on day 6. Mice were placed on a rod rotating at 12 rpm, and the time until the mouse falls from the rod was measured. Each mouse was subjected to six trials per day with a 15-minute inter-trial interval in the training and test phases. We calculated the average value of a set of measurements. The apparatus was routinely cleaned with water and ethanol following each session. We also examined the effects of antipsychotic drugs, i.e., haloperidol and clozapine, in this experiment. Mice were administered vehicle (1% carboxymethyl cellulose-saline, i.p.), haloperidol (0.03 mg/kg, i.p.), or clozapine (1.0 mg/kg, i.p.) every day 30 minutes before the rotarod test.

The other behavioral experiments were performed as described below. The Y-maze test was performed as described previously^52^. Each arm was 40 cm long, 12 cm high, 3 cm wide at the bottom, and 10 cm wide at the top. The arms converged in an equilateral triangular central area that measured 4 cm at its longest axis. Mice were placed in the central area of the apparatus and allowed to move freely during an 8-minute session. The arm entries were sequentially recorded. Alternation was defined as multiple entries into three different arms (A, B or C) on overlapping triplet set. The percent alternation was calculated as the number of alternations divided by the number of total arm entries minus 2 multiplied by 100. Spontaneous alternation (%), defined as successive entries into the three arms on overlapping triplet sets, was associated with the capacity of short-term memory.

The elevated plus maze was constructed and conducted as previously described^53^ with minor modifications. The apparatus was made of plastic material and was elevated to a height of 50 cm above the ground. Each arm of the plus maze was 16 cm in length and 10 cm in width. Additionally, the closed arms had wall enclosures that were 20 cm high. The central platform was a square measuring 10 × 10 cm. The light intensity around the maze was set at 100 to 120 lx. Mice were placed on the elevated plus maze for 5 minutes. They were placed on the elevated plus maze facing the open arm opposite to the experimenter. The number of entries and time spent in the open and closed arms were recorded over the entire duration of the test.

The novel object recognition test was performed as described previously^54^ with minor modifications. In habituation session, mice were placed in an apparatus (30 × 30 × 35 cm) for 3 days. During the training session, two novel objects were placed in the apparatus and the mice were allowed to explore each object for 10 minutes under a moderate light condition (15 lx). The time spent exploring each object was recorded. The preference index was calculated as the ratio of time spent exploring one of the objects to the total exploration time. For the test sessions, mice were placed back into the same apparatus 24 hours after the training session, one of the familiar objects used during training was replaced by a novel object, and the mice were allowed to explore each object (familiar and novel object) for 5 minutes. The preference index in the test session was defined as the ratio of the amount of time spent exploring the novel object over the total time spent exploring the two objects, was used to measure cognitive function.

The PPI test was performed as described previously^55^. After mice were placed in the chamber under a moderately bright light condition (180 lx; San Diego Instruments, San Diego, CA, USA), they were habituated for 10 minutes in the presence of background white noise (65 dB). The mice received three kinds of trials (10 startle trials, 10 no-stimulus trials, and 40 PPI trials). The inter-trial interval ranged between 10 and 20 seconds, and the total session lasted 17 minutes. The startle trial included a single 120-dB white noise burst lasting 40 milliseconds. The PPI trials consisted of a prepulse (20-millisecond burst of white noise at an intensity of 69, 73, 77, or 81 dB) that was followed, 100 milliseconds later, by the startle stimulus (120 dB, 40 milliseconds of white noise). Each of the four prepulse trials (69, 73, 77, or 81 dB) was carried out 10 times. After each of the four prepulse trials, mice received sixty different trials pseudo-randomly, ensuring that each trial was carried out 10 times and that no two consecutive trials were identical. We measured the resulting movement of the animal in the startle chamber for 100 milliseconds after startle stimulus onset (sampling frequency, 1 kHz), rectified, amplified, and fed into a computer, which calculated the maximal response over the 100-millisecond period. The basal startle amplitude was defined as the mean amplitude of 10 startle trials. The PPI was defined as 100 × [1 − (PPx/P120)]%, in which PPx was the mean of 10 PPI trials (each of the four prepulse trials; PP69, PP73, PP75, or PP80) and P120 was the basal startle amplitude.

### *In vivo* microdialysis

Mice were anesthetized with tribromoethanol (Avertin; 200 mg/kg, i.p.) and a guide cannula (AG-6, Eicom Corp., Kyoto, Japan) was implanted in the NAc (+1.5 mm anteroposterior, +0.8 mm mediolateral from the bregma, and 4.0 mm dorsoventral from the skull) according to the mouse brain atlas. Upon recovery from surgery, a dialysis probe (A-I-6-01; membrane length of 1 mm, Eicom Corp.) was inserted through the guide cannula and perfused with artificial cerebrospinal fluid (aCSF; 147 mmol/l NaCl, 4 mmol/l KCl, and 2.3 mmol/l CaCl_2_) at 1.0 µl/minute flow rate. We collected outflow fractions every 20 minutes. After the collection of baseline fractions, mice were treated with METH (2 mg/kg, i.p.) or high potassium-containing aCSF (60 mM; isomolar replacement of NaCl with KCl), the latter of which was perfused for 20 minutes through the dialysis probe. We analyzed dopamine levels in the dialysates using a high-performance liquid chromatography system (HTEC-500, Eicom Corp.) equipped with an electrochemical detector^56^.

### Immunohistochemistry and Golgi staining

*Reln*^*rl-Orl/*+^ and WT mice were deeply anesthetized with tribromoethanol (Avertin; 200 mg/kg, i.p.) and transcardially perfused with ice-cold 0.1 M phosphate buffer (pH 7.4) followed by 4% paraformaldehyde in phosphate buffer. Their brains were removed and incubated with 4% paraformaldehyde in phosphate buffer overnight. For cryoprotection, brains were incubated with 20% and 30% sucrose in phosphate buffer for 1 day each at 4 °C, embedded in Tissue-Tek O.C.T. compound (Sakura Finetech, Tokyo, Japan), and then stored at −80 °C. Brains were cut into 20-µm-thick coronal sections on a cryostat (CM3000; Leica Microsystems GmbH, Wetzlar, Germany), and free-floating sections were used for TH immunohistochemistry. Brain slices were incubated with blocking solution (5% normal goat serum and 0.3% Triton X-100 in phosphate-buffered saline) for 30 minutes, then incubated with a mouse anti-TH antibody (1:200, Merck Millipore, Darmstadt, Germany) at 4 °C for 24 hours with constant shaking. Sections were incubated with peroxidase-labeled polymer at room temperature for 1 hour, and the reaction was visualized using 3,3′-diaminobenzidine (EnVision Kit, Dako, Tokyo, Japan).

Golgi staining was performed using the FD Rapid Golgi Stain Kit according to the manufacturer’s protocol (FD NeuroTechnologies, Ellicott City, MD, USA) and a previous study^57^. Brains were cut into 80-µm-thick coronal sections on a cryostat (CM3000; Leica Microsystems GmbH, Wetzlar, Germany). The images of pyramidal neurons located in layer II/III of the mPFC were obtained using bright-field microscopic (Keyence, Osaka, Japan) at 100× magnification. Only fully impregnated neurons displaying dendritic trees without obvious truncations and isolated from neighboring impregnated neurons were retained for analysis. The quantification of spine density was limited to dendrites 50 to 200 µm from the soma. Spine density was calculated as the number of spines per 10 µm of dendrite length. All dendrites and spines within images were traced using Neurolucida software (MicroBrightField Bioscience, Williston, VT, USA) and analyzed using NeuroExplorer (MicroBrightField).

### Real-time reverse transcription (RT)-PCR

*Reln*^*rl-Orl/*+^ and WT mice were decapitated and their brains were removed. The NAc and midbrain were immediately dissected on ice. Total RNA was isolated using the RNeasy Mini Kit (Qiagen, Hilden, Germany). Residual DNA was removed using the TURBO DNase-free Kit (Invitrogen, Carlsbad, CA, USA). Isolated RNA was converted into cDNA using the Super Script III First-Strand Synthesis System (Invitrogen) for RT-PCR. Quantitative real-time RT-PCR was performed using a 7300 Real-time PCR System (Applied Biosystems, Foster City, CA, USA) in a reaction mixture with a volume of 25 µl containing 12.5 µl of Power SYBR Green PCR Master Mix (Applied Biosystems), 0.5 to 1.0 µg of cDNA, and 0.5 µM primers for each of the target genes. All reactions were performed in duplicate with or without a template control, as well as RT samples. The PCR cycling conditions were as follows: 50 °C for 2 minutes, 95 °C for 10 minutes, and then 60 cycles of 95 °C for 15 seconds and 60 °C for 1 minute. The primers used are shown in Supplementary Table S2. Data were analyzed by the 2^−∆∆CT^ method^58^. Mouse glyceraldehyde-3-phosphate dehydrogenase (*GAPDH*) was used as an internal reference housekeeping gene.

### Statistical analysis

All data are expressed as the mean ± standard error of the mean (SEM). A one-way, two-way, or three-way ANOVA with or without repeated measures was used, followed by Tukey’s test when F ratios were significant (*p* < 0.05). The statistical significance of differences between two groups was assessed using the Student’s *t*-test.

## Electronic supplementary material

Supplementary Information
