# Supplementary material for: Genetic and animal model analyses reveal the pathogenic role of a novel deletion of RELN in schizophrenia
Source: Sci Rep. 2018 Aug 29;8:13046. doi: 10.1038/s41598-018-31390-w (PMC6115412; doi:10.1038/s41598-018-31390-w)
Supplement: Supplementary file 1 — Supplementary Information [file 41598_2018_31390_MOESM1_ESM.docx]

**Genetic and animal model analyses reveal the pathogenic role of a novel deletion of *RELN* in schizophrenia**

Akira Sobue^1,#^, Itaru Kushima^2,3,#^, Taku Nagai^1#^, Wei Shan^1#^, Takao Kohno^4^, Branko Aleksic^2^, Yuki Aoyama^1^, Daisuke Mori^2,5^, Yuko Arioka^2,6^, Naoko Kawano^7^, Maeri Yamamoto^2^, Mitsuharu Hattori^4^, Toshitaka Nabeshima^8,9^, Kiyofumi Yamada^1^, Norio Ozaki^2^*

^1^Department of Neuropsychopharmacology and Hospital Pharmacy, Nagoya University Graduate School of Medicine, Nagoya, Aichi, Japan

^2^Department of Psychiatry, Nagoya University Graduate School of Medicine, Nagoya, Aichi, Japan

^3^Institute for Advanced Research, Nagoya University, Nagoya, Aichi, Japan

^4^Department of Biomedical Science, Graduate School of Pharmaceutical Sciences, Nagoya City University, Nagoya, Aichi, Japan

^5^Brain and Mind Research Center, Nagoya University, Nagoya, Aichi, Japan

^6^Center for Advanced Medicine and Clinical Research, Nagoya University Hospital, Nagoya, Aichi, Japan

^7^Institutes of Innovation for Future Society, Nagoya University, Nagoya, Aichi, Japan

^8^Advanced Diagnostic System Research Laboratory Fujita Health University, Graduate School of Health Sciences, Toyoake, Aichi, Japan

^9^Aino University, Ibaraki, Osaka, Japan

#These authors contributed equally to this work.

*Corresponding author: Norio Ozaki, M.D. Ph.D., Department of Psychiatry, Nagoya University Graduate School of Medicine, 65 Tsurumai, Showa, Nagoya 466-8550, Aichi, Japan.

Phone: +81 52 7442282, FAX: +81 52 7442293

E-mail: ozaki-n@med.nagoya-u.ac.jp

**Figure S1.** T1-weighted MRI shows atrophy of the left cerebral hemisphere, particularly in the frontal and parietal lobes. There are low-intensity areas (arrows) in the right cerebral peduncle and left basal ganglia.

**Figure S2.** Western blotting analysis of RELN in the serum of SCZ patients. Asterisk represents non-specific bands and serve as internal loading controls. This blot is the full-length blot of Fig. 1c.

**Figure S3.** Performance of *Reln^rl-Orl/+^* mice in the elevated plus maze test (a), Y-maze test (b), novel object recognition test (c), prepulse inhibition tests (d), and MK801-induced hyperlocomotion (e). Data represent the mean ± SEM (n = 15 for WT mice and n = 19 for *Reln^rl-Orl/+^* mice in Fig. S3a; n = 23 for WT mice and n = 28 for *Reln^rl-Orl/+^* mice in Fig. S3b; n = 13 for WT mice and n = 19 for *Reln^rl-Orl/+^* mice in Fig. S3c; n = 20 for WT mice and n = 25 for *Reln^rl-Orl/+^* mice in Fig. S3d; n = 14 for saline-treated WT mice and n = 20 for saline-treated *Reln^rl-Orl/+^* mice; and n = 13 for MK801-treated WT mice and n = 21 for MK801-treated *Reln^rl-Orl/+^* mice in Fig. S3e). **p* < 0.05, significantly different from WT mice.

**Table S1. Results of psychological tests in the SCZ patient with the exonic deletion of *RELN.***

| Function | Task Item | Patient Score | Compared to the Norm | Compared to the Average of SCZ Patients |
| --- | --- | --- | --- | --- |
| Screening MMSE | MMSE full score | 22/30 | Impaired* | Average |
| Estimated Intelligence | JART full-scale IQ | 85 | Below Average** | Below Average* |
| Verbal Memory | BACS #1-sum | 11/75 | Impaired** | Below Average* |
|  | BACS #2 | 7/28 | Impaired** | Below Average* |
| Language | BACS #4-1 | 13 words | Impaired* | Average |
|  | BACS #4-2, 3 | 8 words | Impaired** | Below Average* |
| Mental Speed | BACS #3 | 20/100 (60 s) | Impaired** | Below Average** |
|  | BACS #5 | 23/110 (90 s) | Impaired** | Below Average* |
| Attention and Executive Function | |  | - | - |
|  | Modified Stroop Test | 36.62 s (error 5) | Impaired** | - |
|  |  | 37.85 s (error 20) | Impaired* | - |

*mildly below expectation; **well below expectation.

MMSE: The Mini-Mental State Examination; JART: The Japanese Adult Reading Test; BACS: The Brief Assessment of Cognition in Schizophrenia.

**Table S2. Real-time RT-PCR primer sequences.**

| Transcript |  | Ref. Seq. ID |  | Forward primer |  | Reverse primer |  | Amplicon size (bp) |
| --- | --- | --- | --- | --- | --- | --- | --- | --- |
| DA-related genes | | | | |  |  |  |  |
| D_1_ (*Drd1a*) | | NM_010076.3 |  | 5'-ATCGTCACTTACACCAGTATCTACAGGA-3' |  | 5'-GGCAGGAACAGCATCCTTATTG-3' |  | 100 |
| D_2_ (*Drd2*) | | NM_010077.2 |  | 5'-GTCCTGTCCTTCACCATCTCTTG-3' |  | 5'-CGAGACGATGGAGGAGTAGACC-3' |  | 102 |
| DAT (*Slc6a3*) | | NM_010020 |  | 5'-TCCTGGAACAGCCCCAACT-3' |  | 5'-TGTGGTCCCAAAGGTGTCGTT-3' |  | 83 |
| GABA-related genes | | |  |  |  |  |  |  |
| α_1_ (*Gabra1*) | | NM_010250 |  | 5'-CAAGAGCAGAAGTTGTCTATGAGT-3' |  | 5'-GCACGGCAGATATGTTTGAATAAC-3' |  | 215 |
| α_2_ (*Gabra2*) | | NM_008066 |  | 5'-GCTACGCTTACACAACCTCAG-3' |  | 5'-GACTGGCCCAGCAAATCATACT-3' |  | 115 |
| α_4_ (*Gabra4*) | | NM_010251 |  | 5'-AGAACTCAAAGGACGAGAAATTGT-3' |  | 5'-TTCACTTCTGTAACAGGACCCC-3' |  | 118 |
| β_2_ (*Gabrb2*) | | NM_08070 |  | 5'-AACTACATCTTCTTTGGGAGAGGA-3' |  | 5'-GGTCCATCTTGTTGACATCCAG-3' |  | 106 |
| γ_2_ (*Gabrg2*) | | NM_008073 |  | 5'-ACTTCTGGTGACTATGTGGTGAT-3' |  | 5'-GGCAGGAACAGCATCCTTATTG-3' |  | 147 |
| GAD67 (*Gad1*) | | NM_008077.5 |  | 5'-ATACTTGGTGTGGCGTAGC-3' |  | 5'-AGGAAAGCAGGTTCTTGGAG-3' |  | 147 |
| GAPDH (*Gapdh*) | | NM_008084 |  | 5'-CAATGTGTCCGTCGTGGATCT-3' |  | 5'-GTCCTCAGTCTAGCCCAAGATG-3' |  | 124 |
